# Congenital Luxations

**Published:** 1846-04

**Authors:** 


### ART. VI.

*Die angeborne Verrenkungen.* Mit zwei Tafeln. Von LUDWIG JOSEPH MELICHER, Doctor der Medicin und Chirurgie, &c. &c.—*Wien,* 1845.

*Congenital Luxations.* With two Plates. By LUDWIG J. MELICHER.—*Vienna,* 1845. 8vo, pp. 220.

LUXATIONS of the different articulations of the body appearing at birth is a subject which, till within the last few years, has but little occupied the serious attention of the profession either at home or abroad, and the work before us is, we believe, the first monograph on the topic which has been offered to the public. The theme we need scarcely say is far from exhausted, and yet a great store of facts has been accumulated with which it behoves every well-informed medical man to be acquainted. With the execution of the treatise before us we confess we have been disappointed. It is divided into two sections: the former occupied with what the French call *generalities*; the latter with details; and yet from want of due elaboration,—not to say more—there is a lack of distinctness, and an amount of repetition, which is vexatious. The subject however being handled *ex expresso,* and didactically, proves decidedly suggestive; and though we may not closely follow the plan of the author, we shall endeavour to condense the information he communicates, and add such elucidations from other quarters as suggest themselves.

And let it not be supposed that this is a subject which is more calculated to excite curiosity than to instruct the practitioner. Far from it. Cases are neither of rare occurrence, nor of trifling moment.

"The frequency of these luxations," says M. Melicher, of one variety only, "is greater than is generally supposed. Since the year 1826 when Baron Dupuytren first directed attention to the subject, 180 cases, in various joints, have been recorded. In the course of 18 years this eminent surgeon had seen 26 cases, and M. Guerin had met with 30. Heine had witnessed 11 cases of congenital luxation of the femur, Chelius 9, and I have seen 6. Mr. Smith of Dublin records 5 cases in the shoulder-joint, Adams and Cruveilhier have seen it in the elbow-joint, whilst various authors have noticed luxations of other joints, as will be stated in the sequel."

And as to its importance,

"I have known," said Dupuytren, "many individuals affected with *original* luxation, by sheer mistake of diagnosis, confined to bed for years! I have known others subjected to remedies without number,—to blistering, leeching, cauteries, moxas, &c. &c. I remember one poor girl, who had moxas applied no fewer than twenty-one times, without the useless and barbarous treatment, of course, producing the slightest benefit."

Nurses, again, are in this way often subjected to great injustice, and are most unworthily accused, by heart-broken parents, of having carelessly, it may be barbarously, lamed their children for life; annoyances, these, which competent knowledge and intelligence of the medical attendant would at once have anticipated or removed. Although for ages the subject has been involved in obscurity, it has recently, by the rapid advance of science, been greatly illustrated; and though much yet remains to be done, an account may be supplied not devoid of interest, and which may enable the practitioner to encounter and dispose of the cases he meets, with comfort to himself, and satisfaction to his patient.

Observation and experience have confirmed, what a moment's reflection would suggest to every intelligent practitioner, namely, that luxations appearing at birth, may arise from various and different causes. 1. Considering the nature of the process of parturition, no one can be surprised that the several forces which are then applied to the infant coming into the world, should in some instances lead to dislocation of one or more joints. True, the injury to the limb may not always be of the nature of a luxation, but frequently it is so. These kinds of cases it is evident, though occurring at birth, are not entitled to rank properly as congenital luxations. Hence they should receive a preliminary consideration, because as bearing on diagnosis and practice, they must not be overlooked. They belong to the section of the acquired and violent luxations of our author, *Luxationes acquisitæ violentæ*. As produced during the process of parturition they may appropriately be designated as obstetric luxations, *L. obstetricæ*.

The remaining species as often, at all events, occurring during fœtal life, may well be classed under the category of congenital, *Luxationes connatæ vel congenitæ*. They are very different however in their origin and early history. 2. The joints in the fœtus are, more or less, liable to those complaints which occur in these parts in after life. Of these diseases three have been specified as frequently producing dislocation; namely, first, *morbus coxarius*, or coxalgia; secondly, *hydrathosis*, or an increased secretion and accumulation of the synovial fluid; and thirdly, a hypertrophy of the vascular and fatty cellular structure, which has been named the Haversian gland, which mechanically may dislodge the head of the bone from its socket. Diseases of this character may occur in several joints, though they certainly have been noticed more frequently in the hip joint than in the others. These together go to form that species of disorder which has been denominated spontaneous luxation, *L. spontanea, consecutiva*. 3. It would appear that frequently the immediate dynamic cause of the disorder is an irregular action of the muscular apparatus, excited by a derangement in the nervous system,—the true efficient cause,—a species this which may conveniently be designated functional luxation, *L. functionalis*. 4. And fourthly, one of the most aggravated, if not the most common species of this affection, is a complaint which has its origin, not so much from a morbid, as from a defective action. It appears to arise from what, in general terms, may be denominated arrested development of the joints, wherein the beautiful apparatus of these parts is more or less defective and incomplete; the consequences of which will at once be evident. This species constitutes the *L. originalis*, the *L. originelle*, and congenital malformation of Dupuytren. All these species, moreover, may and do occur in very different degrees; and hence there are specimens of *complete* and *incomplete* luxation. Under the head then of *obstetric* luxation, *spontaneous, functional,* and *original,* we shall arrange such remarks as our space allows. The two former need not occupy us long.

1. OBSTETRICAL LUXATION. We commence with the obstetric variety of luxation as the simplest, and probably the most familiar of all. And here, in reference to those not unfrequent blunders to which we have adverted as described by Dupuytren, we may allude to a rule, applicable to all the species, noticed by our author as observed in the great Maternity Hospital at Vienna; according to which the child, when undergoing its first ablution, is minutely examined by the nurse who performs the duty, with the special view of ascertaining that nothing abnormal appears in any part of the frame. If anything of the kind is discovered, it is noted at the time, and all becoming attention is subsequently bestowed upon it. The *localities,* according to M. Melicher, in which luxation from violence may occur, are to be found in every free joint of the body; but they are principally observed at the shoulder, elbow, ribs, hip, and ankle.

With the view of throwing light on the liability of the different joints to luxation, and the causes and circumstances in which it is most apt to occur, the author made numerous experiments on the dead subject with the object of inducing artificial dislocation, and this where opportunity offered, where the child was in utero, as well as out of it.

Accordingly he experimented upon the bodies of children, respectively of the ages of seven, eight, and nine months, and found that the disposition to luxation in them depended upon the incomplete condition of the surfaces of the joints. At the earlier of these periods of fœtal life, the free (arthrodial) joints are barely indicated, and in the latter months there is nothing more than a superficial excavation. In the humerus, for example, there is a wide disproportion between the size of the head of the bone, and the cavity in which it lodges. Besides the condition of the bones and cartilages, the natural laxity of the other component parts of the joints, of the ligaments, tendons, and muscles, may all be regarded as predisposing causes of the accident.

The author instituted another series of experiments upon children who had arrived at the full time; in relation both to the humerus and the femur. He found luxation of the humerus could be most easily accomplished towards the anterior edge of the scapula; and to be more difficult in the directions inwards and forwards into the subscapular fossa, and outwards and backwards into the fossa infraspinata.

Again, as regards the hip-joint, he found that luxations upwards and outwards are the most difficult to superinduce. He succeeded only in two cases out of thirty, in which he attempted to produce this luxation,—the epiphyses in many cases giving way. In this joint the luxation downwards and backwards into the ischiatic notch is most readily effected, though even this is no easy task. M. Melicher never succeeded in producing dislocation downwards and inwards into the foramen ovale, or upwards and forwards upon the pubis. From these experiments, the author believes that, in violent luxations, and probably in others, the head of the femur usually leaves the acetabulum at its anterior and inner edge, and is placed between this spot and the ischium, and then slides backwards; so that luxations during fœtal life, do not take place downwards and backwards into the ischiatic notch, but occur upon its outer edge, and thus the head is either by degrees, or suddenly, shifted from the external edge of the acetabulum by a second dislocation upwards and outwards, into the fossa iliaca externa, and there becomes fixed; an effect which is produced by the position of the extremities of the fœtus in utero; and from their movements, by the contraction of the muscles, especially of the glutei moving the head of the bone upon the external edge of the acetabulum.

But we shall not dwell longer on these details; and may here, in a few words, dismiss the obstetric variety of luxation, which, as we have said, does not accurately belong to the subject of congenital dislocations. The *causes* most likely to operate, enumerated by our author, are irregular and unwonted contraction of the uterus; irregular and unwonted position of the child, more especially of one or other of its extremities, faulty contractions of the maternal pelvis, from mollities ossium, &c. &c. Capuron believes that it may occur in cross births, from the traction used in the inguinal region, by the fingers. Upon six trials of this kind of delivery, effected upon women who had died undelivered, the child being also dead, M. Melicher found that luxation was produced twice, each in one side of the pelvis. This, however, was effected not by the finger but by the crotchet.

The *diagnosis* in this variety of luxation cannot with an average share of attention be very difficult. A degree of external injury must necessarily exhibit itself, if not at the moment, yet within a few hours of the child's birth, the soft parts being implicated not less than the articulation. In fact, however, though there may be present both the external marks of violence, and the symptoms of deeper-seated injury, it will still remain to be ascertained whether the mischief arises from dislocation, or from fracture. This latter accident, it would appear, from M. Melicher's experiments, is the more likely of the two, and will require to be treated upon the common principles for such cases. Respecting cases of luxation, artificially induced, M. Melicher observes, "once dislocated, the head of the bone remained out and was replaced only with the greatest difficulty." In eighteen such cases he only succeeded in effecting reduction three times; once at the shoulder, and twice at the hip. He contends, that the task will prove still more difficult in the living subject, but this seems more than doubtful; and we question indeed, whether such experiments elucidate the subject. Could the parts once be replaced, which, with sufficient care, should certainly be accomplished, the natural action of the muscles and elasticity of the surrounding parts, would greatly contribute to keep all right. The *prognosis* in this variety and the *treatment* must evidently be regulated by the common rules of surgery, and without doubt with a fair prospect of success.

II. SPONTANEOUS LUXATION. Nor need the species of *spontaneous* luxation occupy us long. Every day's experience is teaching that many of those diseases we are familiar with in the adult, are apt to show themselves in the fœtus; and the modifications they thus undergo though curious, are such as the first principles of the science would lead us to anticipate. Diseases of the joints form no exception to this remark, and they, or rather their consequences, form the spontaneous luxations, *L. spontaneæ, consecutivæ,* of our author.

Much still requires to be done in this part of the subject, the information hitherto collected being far from satisfactory. In the way of illustrating the disorder, we shall confine our remarks to the observations which have been made regarding the hip-joint. Three diseases have been enumerated as occurring here, tending to dislocation, and apparently not without cause; namely, the common hip-disease, *coxalgia,* hydrarthrosis, increased synovial secretion, and hypertrophy of the cellular and fatty apparatus within the joint.

*Coxalgia.* Hip-disease is generally assigned as a cause of congenital luxation by writers on these complaints; though we suspect from the general obscurity which has heretofore enveloped the subject, more frequently than was meet. Thus two cases of this sort are given by Albers in his 'Prize Essay on Spontaneous Lameness,' where the symptoms of coxalgia were present, but where M. Melicher suspects that the disease did not play so important a part as is assigned to it; and M. Ficker in his 'Prize Essay' has corresponding observations. Our author states that he has not discovered any well-authenticated case, where an infant came into the world with a spontaneous luxation, the result of morbus coxarius having reached its latter stage; though he has himself witnessed instances in which the disease had commenced in new-born infants, had also made some progress, and was apparently approaching to this melancholy consummation. It is, moreover true, that for the first few months of existence, the occasions of judging of the existence of the disease, whether arising from this particular cause, or from any of the others we have yet to advert to, are far from satisfactory, so that there is scarcely any possibility of detecting them. Medical men, moreover, are often not consulted till the time when the child was expected to have walked is passed, and the anticipations of the parents have been disappointed. Attention to the past history of the case, and a minute examination of the affected parts, can then scarcely fail to guide the judgment of the well-informed practitioner. The diagnostic marks, however, for this purpose, will be best enumerated when the other species of the disease have passed under review, and will, till then, be accordingly postponed.

*Hydrarthrosis.* The principal object of a late memoir on congenital luxation, published by M. Parise, is to demonstrate that this latter affection may arise from an anormal secretion of synovia, and also from a diseased state of what has been called the Haversian gland. An illustrative case of each of these disorders may stand in the place of more lengthened observations. M. Parise states that when he was house-surgeon of the Hôpital des Enfans Trouvés at Paris, he examined the joints of 332 new-born infants. Amongst these, he found only three in which congenital luxation existed from this cause; and the appearances were so similar in all, that the recital of one suffices for the exposition of the others. The subject which afforded this opportunity of examination, was an infant ten weeks old at the time of its death. It had been very much out of health generally. The great trochanters were found further apart than usual, and nearer to the crest of the ilium; the heels were approximated, and the toes turned outwards. On both sides the head of the femur was found partially luxated upwards and outwards, resting on the crest of the acetabulum, where it had formed a slight depression for itself, which communicated with the normal one of a large size. The Haversian gland was not hypertrophied. The double cavity was filled with synovia, which flowed freely when the capsule was opened. The head of the femur and the trochanters were well formed, and the round ligament was long and on the stretch. The capsule was still entire, and the pelvis otherwise in a healthy state. (Parise, Arch. Gén. de Méd. t. xiv, p. 439.)

*Hypertrophy of the Haversian gland.* Several cases of luxation from this cause have now been recorded. Paletta gives one, and as illustrating the disease we subjoin a case. A male child, aged six days, died at the Hôpital des Enfans Trouvés, and exhibited a deformity in both hip-joints, which was found to be owing to a cause alike in both. The pelvis, femur, and surrounding muscles were normal. The head of the femur, slightly depressed backwards, did not correspond with the central axis of the acetabulum. This cavity was oval-shaped, having its larger extremity looking upwards and outwards. The deeper parts were filled with a small tumour of a crimson colour, whose texture was uniform, and of the consistence of lard. This tumour was evidently formed of an enlargement of the cellular and adipose structure, called the Haversian gland, which was thickened to the extent of about two lines, and which extended over a considerable portion of the cartilaginous surface of the cavity; and between these parts there was a whitish pseudo-membrane. At the first view the acetabulum appeared single and much inclined upwards; but, upon narrower inspection, a salient line was found to separate the upper and outer third, from the two lower and internal ones. The head of the femur was lodged in the former of these, and was displaced from the latter by the morbid growth. The round ligament was longer than usual, whilst the capsule appeared natural. From the appearances it was judged that very speedily the luxation would have been complete. (Parise, l. c. p. 446.)

The natural progress and termination of some of the varieties of this species, tedious and aggravated, occurring in vitiated constitutions, is to a most distressing consummation. This is well shown in a case which we copy from M. Melicher's pages. The most distressing termination, he remarks, of these cases, is complete anchylosis of the luxated bones, more especially the thigh-bone and the ilium. Thus, I had a patient, a boy, whose parents assured me that he came into the world labouring under the disease, the consequence, as his mother supposed, of an accident she received in the seventh month of pregnancy, when she was thrown from a carriage and fell upon the abdomen. The child was born at the natural time, and was weakly. It was sent to the country, and there somewhat recruited. When seven months old it was remarked, that the lower extremities were not in their natural position; a circumstance which appeared inexplicable, as the child had never been out of his parent's sight. At a later period it was observed that the child could not move its lower extremities at all. A variety of treatment was then adopted, but without benefit, so that latterly it was abandoned. When seen by M. Melicher, the boy was 14 years of age. All the symptoms of congenital luxated femur were present, with this peculiarity, that the femur was united to the ilium by bony anchylosis. The pelvis was broad, and the rami of the pubis widely separated. The trochanters were prominent and lay close upon the ilium; the nates were elongated and much vaulted outwards; no force could move the femur, its head being firmly fixed upwards and outwards upon the external part of the ilium. The thighs were rotated inwards, the knees were closely approximated and almost immoveable. The legs again, extended outwards, as in the talipes valgus, the toes however turning inwards, and the heels outwards.

Notwithstanding the prominence which on this topic has long been paid to the subject of spontaneous luxations, we will not now longer dwell upon it. That prominence, we believe, has in no inconsiderable degree been owing to the very obscurity in which the subject has been enveloped, and which recent researches have gone far to remove. Much loose and general discussion—we had almost said declamation—has long prevailed, which we anticipate before the views we have now to detail will speedily disappear.

III. FUNCTIONAL LUXATION. *L. functionalis.* We must here say a few words in way of apology for introducing a new name for this species of luxation, which is not employed by our author, nor, so far as we know, by any other writer on the subject. We venture on this step, not because we attach any importance to a mere name, but because accuracy of terms is essential to accuracy of thought and discussion; and because much confusion and obscurity have resulted in medical, as in other branches of natural science, from carelessness and inaccuracy in nomenclature. One of the great sections into which the author has divided his subject, is that of acquired luxations, *L. acquisitæ*, and under this title he has arranged the species we are now considering. But this, so far as the nature of the affection is concerned, is anything but a specific distinction, and is calculated far more to perplex than elucidate. Many of our readers must be familiar with the fact, that the term *acquired* has already been appropriated in the cognate subject of club-foot, which, in fact, is only an example of that complaint on which we are now dwelling; and that it has thus been adopted in medical science as directly opposed, and in contradiction, to the term congenital; the former implying that the disease has made its appearance subsequent to birth,—the other that it has had an existence previous to that event. And many moreover must know, that however important one's birth may be in many particulars, in the matter in hand, whether club-foot, or the disease on which we are now dwelling, it is, in truth, of no kind of moment.

But without dwelling longer on this point, we remark, that we can in no way more effectually, or shortly, elucidate this important species of the so-called congenital luxation, than by exhibiting the true pathology of club-foot, or talipes, as lately expiscated; and by applying it to the whole class, corresponding to our present species, of which it is, in truth, nothing more than a specimen. The new views obtained in talipes must be ranged among the most brilliant improvements of modern surgery; and the light they throw on this hitherto obscure subject, is of the most satisfactory nature.

The different opinions which have hitherto been current regarding club-foot—and the remark equally applies to what has been called congenital luxations—are such as the following: 1st. That the original formation of the bones principally implicated has been unnatural and incomplete. 2d. That the bones, though originally formed perfect, became injured and distorted by causes independent of the formative process; as by the pressure of the fœtus, occasioned by irregular action of the uterus, anormal position of the fœtus, by blows, concussions, &c. &c. And 3d. That whatever may have been the condition of the bones previously, the act of walking displaced and injured them. (See Little on Club-Foot, p. xxii.) Upon these various and discordant views, physiology and pathology have recently shed their light, and brought order out of confusion. Scarpa was one of the first to elucidate the important truth that in talipes none of the tarsal bones were actually dislocated; but, in the anormal state of the ankle, undergo distortion of their axes, more or less extensive. Benjamin Bell, Boyer, and Jörg, again taught that irregular muscular action had an important influence over the disease, whether produced by the debility and paralysis of one set, or the over action and spasm of their antagonists, or from these causes conjoined. Finally, Rudolphi completed the true pathological views by demonstrating that congenital talipes, and the analogous disorders, arise from the disordered influence of the nervous energy of the muscles in the fœtal state, whereby irregular action is superinduced, and distortion and deformity result. Delpech's interesting cases had previously clearly demonstrated, that all these changes could be effected after birth, and even in adult life not less than in utero; and the genius of Thilenius, Stromeyer, Dieffenbach, and we must add our countryman Dr. Little, brought all these facts to bear upon practice. So that this irregular action, and its distressing effects, whether evinced in the most delicate muscle of the body, as in one of the recti of the eyeball, or in the strongest, as in the tendo-achillis—whether in the deformity of squinting or of club-foot—can be removed by an operation not more simple than effectual.

The application of the principles to which we have just adverted, to the subject of "congenital luxations," is too apparent to require any lengthened demonstration.

Instead of this species of luxation being considered as it really is, a mere distortion of parts originally well-formed, (whether occurring in the fœtal state or subsequently,) the popular notion is, that it depends upon some deficiency of the parts, some malformation, monstrosity, or arrest of development. The refutation of this, alike short and complete, is the scalpel, which shows the bones and other apparatus, somewhat distorted indeed, but otherwise normal.

The next cause assigned for this kind of "congenital luxation," and one now the most popular, is that it follows external injuries to the part during fœtal life; as from blows and shocks, abnormal action of the uterus, or malposition of the fœtus. A moment's consideration of the circumstances of the fœtus,—a solid body floating in fluid, and in other circumstances peculiarly calculated to prevent any such effect,—might, on mere mechanical principles, have demonstrated that such an effect could not be produced. But the most direct answer to this hypothesis is, that these luxations take place after birth; and even sometimes in adult years, as well as in utero. It is ignorance of this fact that has thrown so much obscurity and confusion over the whole subject.

The true origin of the phenomena of these so-called "congenital luxations,"—what we venture to designate functional luxation,—is to be found in some cause resident within the organism of the fœtus. This is a point of so much interest, that we cannot better illustrate it than by a few sentences from Dr. Little's excellent work on Club-foot.

"A child during the progress of dentition, is observed to drag one leg, sometimes both after him; in other words does not possess the full voluntary power of moving it; he has a slight limp in his gait, depending on a slight rigidity or contraction of the muscles of the calf, and consequent stiffness or inability of bending the ankle-joint. The former case arises from partial paralysis of some of the flexors of the joint, the latter from spasmodic contraction of the extensors of the foot. The disease advances unchecked by medical treatment, and the result is one or other form of club-foot. In other cases during the period of life when the nervous system is most sensitive, a child debilitated by some violent disorder, or by a succession of infantile complaints, or by croup, or what is called a fit, is said to have lost the use of one or all his limbs, which disorder may be paralytic or spasmodic. This may affect the trunk, giving rise to deformity of the spine, or the shoulder, elbow, hand, producing the deformities known as club-hand, and the scrag-hand, or the fingers; also the lower extremities, the hip, knee, ankle; in fact, every joint of the body. I have treated," says Dr. Little (and we have had such a case under our care), "a child born with spasm of the muscles of the eyes, of the spine, of the adductors of the thighs, and muscles of the calf, producing squinting, partial opisthotonos, rotation of the thighs inwards, and double talipes equinus. And the whole of these affections with the exception of the strabismus, have been almost entirely removed by the use of preparations of iron, calomel, rhubarb, and chalk, with long-continued extension, counter-irritation to the spine, with general friction and champooing."

The efficient cause of these muscular irregularities, which are to be considered as the dynamic cause of the "luxations," resides in the nervous system; either in the nervous centres—the brain or the spinal cord; or the disease exists in some other organ of the body implicating the peripheral parts of the nervous system; for instance, some of the abdominal viscera, the incident nerves of which are morbidly affected; these communicate in the spinal cord, with other filaments—the reflex, or involuntary motor nerves—whereby the muscles of the deranged part are excited to spasmodic action.

Upon this theme we could willingly enlarge, but we forbear; and chiefly because we believe enough has been said to satisfy every intelligent reader of the true nature of this species of the long obscure and perplexed subject of "congenital luxation." The view has all the charm, all the simplicity, and force of truth.

One variety of this species of luxation, as we believe, must not be passed over, as we have not noticed it mentioned by any other writer on the subject, save our author. He calls it *luxatio congenita costarum*, and remarks, that in large hospitals, children are observed to come into the world, emaciated, small, and weak; in many of whom the ribs are more or less compressed, the sternum and abdomen projecting, the spine inclining backwards. In others, several of the ribs, at their junction with the sternum, especially the fourth, fifth, sixth, and seventh, are found strongly projecting, as if broken. There is however no fracture. The appearance is produced in the cartilages connecting the ribs to the sternum. These are distorted, generally at their costal extremity, sometimes at their sternal. In after years, this constitutes what is familiarly known as chicken-breast. This affection may occur simultaneously on both sides, or be confined to one only. Generally, several ribs are thus "luxated," seldom one or two. The deformity is most frequently found in the four under true ribs.

No difficulty need, of course, remain from the name which has so long been imposed upon cases belonging to this important species. They received the name of *luxation* when their pathology was unknown; just as club-foot was held to be a luxation of the ankle-bones, when it was nothing more than distortion. And they were called *congenital*, because, doubtless, they often do occur before birth; although this is far indeed from being an essential attribute.

And this leads to an observation to which we must revert, after treating of the remaining species of these congenital luxations, namely, that frequently, during the first few months after birth, they are not detected by the relatives or attendants. The cause may have operated in utero, but the child at birth is to all appearance in good health. It continues so; and, if the infirmity be in the lower extremities, these not being brought into play in the first months of existence, it is only after these have elapsed that the disease begins to declare itself. Something also must be said in a subsequent page upon the diagnosis. And the powers of the art on this species of the disorder are not to be contemned. They were of purpose alluded to in the most aggravated case detailed in a former page, and are assuredly of a very high order.

But our contracting space reminds us that we must hasten to the last species of congenital luxation, which has to be brought under notice, perhaps the most singular of all; and which, thanks to the improvement of modern pathology, has at length received all necessary elucidation.

IV. ORIGINAL LUXATION. *L. originalis, Luxation originelle* (Dupuytren), *L. congenita,* Claudicatio, Congenital malformation. This fourth species of congenital luxation, though not altogether unknown in this country, has yet so recently been described, and is so far from being familiarly known, that we shall only be doing good service in presenting, in this its natural connexion, a summary account of it. It is undoubtedly the most striking and melancholy species of the four; and cases are even now coming into our hospitals bearing indelible marks that they had previously been misunderstood. It embraces that considerable class, wherein is included the most aggravated cases of lameness and distortions,—of unfortunate cripples, who cannot be seen without exciting heartfelt sympathy and pity.

The original luxation is apt to occur in most of the free joints, as in the shoulder, elbow, and wrist, in the hip-joint, knee, and ankle; and, for very obvious reasons, it is most afflictive in the larger of them. In them also it is unfortunately the most frequent. As our space forbids us to enlarge, and our object is merely to illustrate, we shall confine our attention chiefly to the hip-joint; the nature and effects of the disorder being,

*mutatis mutandis*, substantially the same in the others, so that if understood in this case, the disease will readily be comprehended in them all.

The variety of congenital luxation, we are now to consider, was first accurately explained to the profession nearly twenty years ago, by Baron Dupuytren, whose description for vigour and accuracy has not been excelled; and the most important additions made to the facts stated by him, are those collected by Mr. Adams of Dublin. The characters of this luxation, according to the Baron, are a shortening of the affected member, the ascent of the head of the bone into the external iliac fossa, the projection of the great trochanter, the retractation of nearly all the muscles of the upper part of the thigh towards the crest of the ilium, where they form, round the head of the femur, a kind of cone, whose base is at the ilium, and whose summit is the great trochanter; the almost total denudation of the tuberosity of the ischium deprived of its muscles, the rotation of the limb inwards, and consequent abduction of the heel outwards, and the knee and great toe inwards, an obliquity which increases with advancing years, and as the pelvis increases in size, whence results a tendency in the femurs to cross each other inferiorly, making an acute angle where it is attached to the pelvis, and finally, great emaciation of the lower limb, more especially of its superior part.

The isolated movements of the deformed members are in general very confined, especially those of abduction and rotation. Hence innumerable difficulties in standing, walking, and every other movement in which the limbs play a part.

When the person is standing, we are struck with the want of proportion between the upper and lower parts of the body, the imperfection of the lower limbs, and the attitude of the individual. The trunk is well developed, while the lower limbs are short and slender, as if they belonged to a much smaller person. The diminutive size of their limbs appears the more striking on account of the great breadth of the pelvis, and we are surprised with the projection of the trochanters. Regarding the attitude, we observe that the upper part of the trunk is borne far back, and the spine projected forwards, and very hollow behind; the pelvis is placed on the femurs nearly horizontally, and the unfortunate sufferer does not touch the ground except with his toes.

A person thus deformed, when about to walk, balances himself on his toes, then wholly inclines the upper part of his body towards the member upon which he is about to repose his weight, raises the opposite part from the ground, and then painfully transports his weight to it. In fact, every time that this change is effected, the head of the femur, which receives the weight of the body, is elevated in the fossa of the ilium, the pelvis descends, and all the signs of displacement become more marked on this side, while they become less apparent on the other, till it, in its turn, receives the weight of the frame; and it is by this succession of efforts that the weight of the body is alternately transposed from one limb to the other. The cause of all these painful efforts is most evidently the loose condition of the head of the femur, and its continual displacement upwards and downwards, as it receives, and is freed from, the weight of the frame.

At first view, it appears singular that running and leaping to such a person should be easier than walking. This however is the fact. And for this reason, that in running the energy of the muscular contraction, and the rapid transfer of the weight of the body from one leg to the other, renders the deficiency of the acetabulum, and the looseness of the head of the femur, almost inappreciable. This mode of progression however is so fatiguing to these individuals that they cannot continue it long.

When persons afflicted with this infirmity lie on their back, we are astonished to find that the signs of their infirmity diminish and disappear; which is owing to the circumstance that when thus in repose, the muscles cease to elevate the femurs, and the weight of the upper part of the body, like a cone, presses down the pelvis between the femurs. What authenticates the accuracy of this explanation is, that in this position you can elongate or shorten the limb at will by slight traction, and to an extent of two or three inches, according to the age of the patient, and the extent of the displacement; and all the appearances are at once affected thereby. All these changes are, moreover, effected not only with great ease, but without pain, clearly showing that there is no kind of disease, properly so called, present, and nothing more than a want of the acetabulum and attending alterations.

The *Lusus naturæ* here is so plain as scarcely to require any explanation. According to Dupuytren all the implicated muscles are very much drawn up towards the crest of the ilium, and they are more or less atrophied; some of them being very much converted into mere fibrinous tissue. According to the same authority, the upper part of the femur maintains very much its natural form and dimensions. The acetabulum is either wholly wanting, or is represented by a small osseous projection, in which there is no trace of cartilage, capsule, or rim. Occasionally the round ligament may be seen, but much changed. The head of the femur is found lodged in a cavity analogous to those met with in unreduced luxations, running upwards and backwards. This new cavity, superficial and rimless, is situate on the external iliac fossa, and has limits differing in different cases.

It is worthy of notice, that within Dupuytren's observation this distressing affection occurred but rarely on one side of the pelvis only, and much more frequently upon both. He states that out of twenty-six cases he had witnessed, the luxation was confined to one side only in two or three persons. This experience, however, does not seem to correspond with that of others. Our author states that Professor Chelius had witnessed nine cases, and four of these were on one side only; and Mr. Adams mentions that he had seen the affliction more frequently upon one side than on both. This comes to be an important fact in the matter of diagnosis, and therefore should not be overlooked. It has also considerable bearing on the symptoms and pathology, and should not be disregarded.

When one side only is implicated, remarks Mr. Adams, the weight of the body is thrown nearly wholly on the sound limb, which becomes stronger and larger than usual, while the other becomes more or less atrophied, its circulation languid, and its nervous energy and temperature diminished,—phenomena which are apparent in both limbs when both are affected.

Mr. Adams adds, that when one hip only is abnormal, we find that a lateral curvature of the spine exists, and the bones of the pelvis are atrophied on the malformed side. He also observes that the os innominatum of the deformed side, together with the femur, and other bones of the lower extremity of the same side, are much smaller than the corresponding bones of the sound extremity; and the former, besides being deformed, are in a state of atrophy in circumference and length, while those of the latter are evidently larger, and better nourished than we could expect in such delicate individuals. In a word, there is a compensation growth of the skeleton on the sound side, to make up, as it were, for the deficient growth of the other side; the head of the femur, too, and the acetabulum are both very large, as is the whole of that side of the pelvis.

Another particular in which Mr. Adams's observations differ from those of Dupuytren, and we believe corrects them, is that wherein the Baron remarks that the upper part of the femur very much preserves its form and natural arrangements. According to Mr. Adams there is, on the contrary, a marked change in these particulars. 1st. The neck of the femur, instead of having its axis directed, as it is naturally, upwards and outwards, loses its natural relation to the shaft of the bone, and the axis is directed upwards and almost straightforwards. And 2d. The head of the bone instead of being directed backwards, as in ordinary luxation, on the dorsum ilii, on the contrary, is directed forwards, and placed beside the anterior inferior spinous process of the ilium, while the trochanter major is directed backwards on the dorsum ilii. The following remarks likewise are too valuable to be omitted: "The anterior spines of the ilium, particularly the inferior, we have usually found to be directed very much inwards towards each other,—the external iliac fossa to be much more convex, and the internal to be much more concave than usual; beneath the anterior inferior spine, we notice a deep groove directed outwards. The sub-pubic angle is remarkably obtuse, the rami of the pubis and ischia are very oblique, and the tuberosities of the ischia everted. (Todd's Cyclopædia, vol. ii.)

It should here, as before hinted, be particularly noted that though these appearances are so marked on dissection, and so striking in the adult, yet are they far from being conspicuous in the infant. If called to examine a case shortly after birth, there are no doubt indices of the vicious malformation, such as the great breadth of the pelvis, the projection of the head of the femur, the obliquity of its shaft, &c. &c. But it usually happens that the abnormal conformation, and the infirmities which result from it, do not attract the attention of the parents and attendants till the time when the child should begin to walk, or actually attempts it, and it is in these circumstances only that the surgeon is appealed to. Then it is ascertained that the child has great difficulty in standing, and still more in walking. But it frequently happens that the friends even then shut their eyes against the calamity which threatens, and making their wish the father of their thought, cherish the conviction that the child is backward only as it respects its walking powers, and will not admit for years that there is any distinct infirmity, not until the defect and imperfection in the form and action of the parts have become so conspicuous, that it appears altogether unreasonable to attribute them to any mere retardation of development and action.

All this shows the importance of an accurate *diagnosis* between affections which it has been remarked, in infancy especially, are so analogous in their symptoms, and so different in their origin, nature, and treatment—the diagnosis between this *original* luxation, and each of the three species which have preceded. Great assistance is here procured from the absence of all pain, swelling, abscess, fistula, or cicatrix, which are more or less found in all the others; and also from the fact that the subject of this defect is generally in all other respects in good health. In obstetrical luxations, and other accidents, there are always present the local signs of injury and contusion; in spontaneous, the symptoms of more confirmed disease, both local and constitutional,—a remark which may be extended to functional luxation. The coincident appearance of this deformity on both sides of the body, though not so common, and therefore not so pathognomonic, as supposed by Dupuytren, is yet, where it occurs, of great value. Although there is no pain in cases of original luxation, either about the hip-joint or the knee, yet there is great inability to move, and great fatigue from the attempt. Though there is no morbid swelling about the parts, there is very marked projection of the trochanters, and the soft parts which surround it, and much unwonted movement in them, wholly unknown in the other exhibitions of the disease. The change of form also in this species is very marked, and differs from that which occurs in the others, as is well illustrated by M. Melicher in his 2d plate, (figs. 3, 4, 5, 6, and 7.)

Such then is an illustration of this congenital luxation as it occurs in the hip-joint, apparently its most common seat. It is by no means, however, confined to this articulation; and to Mr. Smith's pen are we recently indebted for a most lucid exposition of its occurrence in the shoulder-joint. He details five cases observed in Dublin; three in which the luxation was sub-coracoidal, and two in which it was sub-acromial. Of the former, two were in the one shoulder, the left, and one in both. Of the latter, one was in the left, the other in the right side. Mr. Smith also discovered unequivocal evidence of the existence of other cases in several museums, and has brought under this category various anomalous cases which flourish in the archives of the science, as perplexing instances of partial and incurable dislocation. In the examples of this congenital disorder the member was nearly useless, in some entirely so. (Mr. Smith's Paper in Dub. Journ. of Med. Science, 1839.) The same disorder has been observed at the elbow- and knee-joint, also in the radius and ulna, and the tibia.

Upon the *cause* of this original luxation we need not enlarge. That it is congenital will be disputed by none; and that it is original, and takes date from the first organization of the articulation, will be conceded, we believe, by all who take the trouble to investigate the case. It appears to be a defect of the organization in the very germ. This hypothesis accounts for the double luxations, not less readily than the single; and moreover for the perfect health, in all other respects, of the new-born infant, and for its thriving for months and years, with this exception. In perfect keeping with this view some physiologists have combined the speculation, that with the local defect in the articulation there is a corresponding lesion in the brain or other part of the nervous apparatus. This agrees accurately with what has been previously stated as the cause of functional luxation; and though there are not wanting grounds upon which this speculation has been based, as it respects the present species, we have now no time to adduce them.

That original luxation should occasionally show itself hereditarily will excite the surprise of none. Commencing, though frequently covertly, with the primal days of infancy, it maintains a steady and obstinate course, becoming rather more aggravated with advancing years, though in a few cases, all things considered, there is a surprising degree of agility. Dr. Maissiat, who communicated the details to Dupuytren, informs us that in one family where the disorder showed itself hereditarily, four individuals, who were related to each other, as aunts and nieces, were respectively of the ages of 70, 80, and 84. The niece had a daughter who suffered under an original luxation in her right femur; and this last individual had four children, two of whom had the same deformity, the one on both sides, the other only on the left side.

It might, at first sight, be apprehended that for a complaint, so serious as that on which we have been dwelling, no remedies would be of any avail; but in many cases, at all events, this is not the case. Not that we speak of cure,—but of relief. Scrupulous attention must be paid to the general health, and double diligence bestowed on the infirm member, by the use of friction, champooing, and gentle traction, whereby its nervous and muscular energy may be augmented. And the palliative remedy, so far as the hip-joint, for example, is concerned, is the accurate and firm fitting of a kind of girdle to the pelvis, which is wadded and furnished with a groove for the head of the femur, so that its movements may be restrained, and much more assurance given to the step, and stability to the frame. What has been so successful in the hip-joint, could not fail in similar cases, with equal ingenuity, to be not less so in the others, and the patients' comfort thereby be much increased.

Thus, then, in reviewing the whole subject of this monograph, we find that the topics it embraces involved, even in our own time, in almost impenetrable confusion and obscurity, with the advance of the science, present entirely a new phase. Regarding congenital luxation as a genus, or natural group, we find that it very readily divides itself into the species of *obstetric*, *spontaneous*, *functional*, and *original* luxation, all having many points of resemblance, and yet each very distinct in its nature from its fellows. Viewed severally that nature is simple, and its treatment clear, and the diagnosis, to a careful and well-informed practitioner, can seldom remain long doubtful. We are far from asserting that the subject is even now exhausted. But sure we are that there is abundant ground for gratulation on account of the advances which have recently been made, including some of the brightest triumphs of modern surgery, gratifying alike to the admirer of his art, and the well-wisher of his kind.

